# Quantitative Assessment of Heart Rate Dynamics during Meditation: An ECG Based Study with Multi-Fractality and Visibility Graph

**DOI:** 10.3389/fphys.2016.00044

**Published:** 2016-02-16

**Authors:** Anirban Bhaduri, Dipak Ghosh

**Affiliations:** ^1^Deepa Ghosh Research FoundationKolkata, India; ^2^C. V. Raman Centre for Physics and Music, Jadavpur UniversityKolkata, India

**Keywords:** meditation, ECG, MF-DFA, visibility graph, fractal

## Abstract

The cardiac dynamics during meditation is explored quantitatively with two chaos-based non-linear techniques viz. multi-fractal detrended fluctuation analysis and visibility network analysis techniques. The data used are the instantaneous heart rate (in beats/minute) of subjects performing Kundalini Yoga and Chi meditation from PhysioNet. The results show consistent differences between the quantitative parameters obtained by both the analysis techniques. This indicates an interesting phenomenon of change in the complexity of the cardiac dynamics during meditation supported with quantitative parameters. The results also produce a preliminary evidence that these techniques can be used as a measure of physiological impact on subjects performing meditation.

## 1. Introduction

The control of thought, attention and emotion is a difficult problem. *Thinking of nothing* or rather ***not*** *thinking of something*, is the most difficult challenge faced by every human being at different stages of life. An accomplished football player, a singer, musician, physicist or a medical surgeon: the performance of all of them depends only on their ability to focus or concentrate. This concentration, in turn, is largely dependent on their ability to resist distraction and controlling of their impulses and emotions. Meditation often involves an internal effort to self-regulate the mind in some way. Meditation, though for a long time was avoided as a scientific theme due to its complex nature and religious connotations, is studied with different neuro-scientific experimental tools, in the recent times. Moreover, it is often said to be influencing stress-dependent diseases such as anxiety, hypertensive disorders, tension headaches etc. It has been an intriguing source of discussion in the recent literature of neuroscience (Lutz, 2008; Grant et al., 2013; Berger, 2014). Meditation has been described as a clinically relevant method for the cure of stress-related problems (Everly and Lating, 2012) and its clinical effectiveness is also established in different research works (Austin, 1998).

In-spite of numerous popular reports, scientific research in the area of assessing potential health benefits of various types of meditation methods, is scanty in the domain of physiologic effects. We propose to analyse the non-stationary cardiac dynamics during meditation quantitatively, with two chaos-based non-linear techniques viz. multi-fractal detrended fluctuation analysis and visibility network analysis.

The rest of the paper is organized as per following. Section 2.1 describing previous works in similar area of research, Section 2.2 describing the non-linear methods in the light of Chaos Theory, Section 2.3 showing how we have calculated the MF-DFA (multi-fractal detrended fluctuation analysis) parameter, Section 2.4, detailing out the visibility graph technique, Section 3.1 describing the data and Section 3.2 discussing the results. The paper ends with discussion and conclusion in Section 4.

## 2. Materials and methods

### 2.1. Earlier works and methods

Probably a first of its kind of analysis of the heart rate dynamics during meditation was reported by Peng and Mietus (1999) measuring the heart beat signals of subjects performing Kundalini Yoga and Chi meditation. In a further work by Peng and Henry (2004), it was suggested that different meditations evoke common effects on the heart rate, supporting the concept of a *meditation paradox* with a variety of meditative techniques producing active instead of quiescent cardiac dynamics.

The findings of Peng was interesting but conventional methods like Fourier analysis were used as techniques for analysis which had been challenged for quite sometime for non-stationary signals.

In any signal the spectrum may cover wide range of frequencies and also includes the spurious harmonics which is generally left unattended during analysis with the conventional methods. As for example Fourier spectral analysis is based on linear super-positions of trigonometric functions. Additional harmonic components, as is common in most natural non-stationary time-series of which heart rate signal is not an exception, may produce a deformed wave profile. Those deformations are the well known consequence of non-linear contributions. Thereby application of conventional and standard methodology to analyse a time series like heart beat where non-stationary and non-linear components induce spurious harmonic components, makes little sense with respect to the results (Sarkar and Barat, 2008; Conte et al., 2009).

In the recent years, we have witnessed a spur of interest in studying complex systems, natural or man-made in the light of rigorous chaos based non-linear methods. A lot of works had been reported where the study of EEG, ECG, or EMG had been done with non-linear methodologies (Acharya et al., 2005; Ahmadlou et al., 2010; Rodriguez-Bermudez and Garcia-Laencina, 2015). Jiang et al. (2013) had studied heart beat signals using visibility graph methods but no quantitative assessment on the change of ECG patterns due to meditation had been analyzed.

In view of this we attempt to quantify the change of heart-beat patterns during meditation with data for Chi and Kundalini Yoga using two most recent and rigorous techniques.

### 2.2. Chaos fractals and non-linear methods

The word fractal was first introduced by Mandelbrot and Ness (1968). Fractal is a geometrical pattern which is repeated at small or large scales to produce self similar irregular shapes or surfaces that Euclidean geometry cannot explain. Such fractal structures can be seen in natural objects such as a fern leaves, trees, snow-flakes and even clouds or galaxies in space. The most important feature of fractals is its property of self-similarity. An arbitrary region of a fractal looks very similar but not necessarily identical to the entire region. Fractals can be classified into two categories: **mono-fractals** and **multi-fractals**. **Mono-fractals** are those whose scaling properties are same in different regions of a system. **Multi-fractals** are complicated self-similar objects, consisting of differently weighted fractals, with different non-integer dimensions. Thus, the fundamental characteristic of multi-fractality is that the scaling properties may be different in different regions. Thereby a single scaling exponent can not describe the dynamics, with the minutest detail. Many natural systems do not show mono-fractal properties and thereby requires a multi-fractal analysis of the data as proposed by Kantelhardt et al. (2002).

Calculation of fractal dimension or rather measuring the self similarity had been a major area in the field study of chaos. Till date there are several methods proposed for measuring Fractal Dimension (FD).

1. Spectral Analysis
2. Rescaled Range Analysis
3. Fluctuation Analysis (FA)
4. Detrended Fluctuation Analysis (DFA)
5. Wavelet Transform Modulus Maxima (WTMM)
6. Detrended Moving Average (DMA) etc
7. Multi-fractal Detrended Fluctuation Analysis (MF-DFA)
8. The latest addition in the above list is PSVG or Power of Scale-freeness of a Visibility Graph.

### 2.3. Multi-fractal detrended fluctuation analysis

Multi-Fractal Detrended fluctuation analysis algorithm was developed for detecting the long-range correlation and the fractal properties in stationary and non-stationary time series (Hausdorff et al., 1996). The first successful application of Multifractal Detrended Fluctuation Analysis (MF-DFA) method was done by Kantelhardt et al. (2002) during the study of the scaling behavior of various non-stationary scale invariant time series. Following are the steps for **Calculation of width of mutifractal spectrum** used here to analyse the heart-beat dynamics.

1. Suppose for each sample signal *x*(*i*) for *i* = 1, 2, …, *N*, is the waveform of *N* time instants. The mean of this time-series is calculated as $\bar{x}=\frac{1}{N}\overset{N}{\underset{i=1}{\sum }}x\left(i\right)$ Then the integrated series is computed as per Equation 1 of Kantelhardt et al. (2002).
  $$\begin{array}{l} Y\left(i\right)=\overset{i}{\underset{k=1}{\sum }}\left[x\left(k\right)-\bar{x}\right],i=1,2,\ldots ,N \end{array}$$
2. The integrated time series is divided into *N*_*s*_ non-overlapping bins (where *N*_*s*_ = *int*(*N*∕*s*) and *s* is the length of the bin) and the fluctuation function is computed. For each *s* the local RMS variation is calculated as function *F*(*s, v*) as per Equation 2 of Kantelhardt et al. (2002).
  $$\begin{array}{l} F^{2}\left(s,v\right)\equiv \frac{1}{s}\overset{s}{\underset{i=1}{\sum }}{\left\{Y\left[\left(v-1\right)s+i\right]-y_{v}\left(i\right)\right\}}^{2},\\ where\;i=1,2,\ldots ,s\;\text{and}\;v=1,2,\ldots ,N_{s} \end{array}$$
  Here *y*_*v*_(*i*) is the least square fitted polynomial of the bin *v*. *y*_*v*_(*i*) is defined as $y_{v}\left(i\right)=\overset{m}{\underset{k=0}{\sum }}C_{k}{\left(i\right)}^{m-k}$, where *C*_*k*_ is the *kth* co-efficient of the fit polynomial with degree *m*. Here we have taken *m* as 1.
3. The *qth* order overall RMS variation for each scale *s*, is denoted by *F*_*q*_(*s*) which is calculated as per Equation 4 of Kantelhardt et al. (2002) as shown below.
  $$\begin{array}{l} F_{q}\left(s\right)\equiv {\left\{\frac{1}{N_{s}}\overset{N_{s}}{\underset{v=1}{\sum }}{\left[F^{2}\left(s,v\right)\right]}^{\frac{q}{2}}\right\}}^{\frac{1}{q}} \end{array}$$
  For our experiment we have calculated *q*-th order RMS variation *F*_*q*_(*s*) for 100 equidistant values of *q* in between the range of −5 to +5.
4. The steps 2 and 3 are repeated and *F*_*q*_(*s*) is calculated for various values of *s*. If the time-series is long range co-related, the *F*_*q*_(*s*) vs. *s* for each *q*, will show power law behavior as below.
  $$\begin{array}{l} F_{q}\left(s\right)\propto s^{h\left(q\right)} \end{array}$$
  If such a scaling exists log_2_(*F*_*q*_(*s*)) will depend linearly on log_2_*s*, where *h*(*q*) is the slope. The exponent *h*(*q*) depends on *q*. Here *h*(*q*) is the generalized Hurst exponent. This *h*(*q*) of MF-DFA is related to the scaling exponent τ_(_*q*) as per Equation 13 of Kantelhardt et al. (2002) as shown below.
  $$\begin{array}{l} \tau_{\left(q\right)}=qh(q)-1 \end{array}$$
5. Multi-fractal signal have multiple Hurst exponents, hence τ_(_*q*) depends non-linearly on *q*. If α is singularity strength and the singularity spectrum is *f*(α), *f*(α) is related to *h*(*q*) as per Equation 15 of Kantelhardt et al. (2002).
  $$\begin{array}{l} \alpha =h\left(q\right)+qh^{\prime }\left(q\right)\text{and}f\left(\alpha \right)=q\left[\alpha -h\left(q\right)\right]+1 \end{array}$$
  The resulting multi-fractal spectrum *f*(α) is an arc where the difference between the maximum and minimum α is called the multi-fractal spectrum width. The width of the spectrum gives a measure of the multi-fractality of the time-series. Greater value of this width indicates presence of greater amount of multi-fractality in the series.

MF-DFA method is a robust tool to perform scaling analysis in case of non-linear time-series. Results obtained by this method turn out to be more reliable in comparison to methods like wavelet analysis, discrete WT, WTMM, DMA, BMA, MDFA, CDFA etc (Kantelhardt et al., 2002; Oswiecimka et al., 2006; Serranoa and Figliola, 2009; Huang et al., 2011). The only limitation of this technique is that it requires longer time-series which is sometimes difficult to obtain from real dataset. In the present work we make comparisons between the multi-fractal spectral width derived from the heart rate signals and discuss the visible differences.

### 2.4. Visibility graph

A radically different but rigorous method named Visibility graph analysis is reported by Lacasa et al. (2008). Recently this method is extensively used over finite time-series data set and has produced reliable result in several domains of science and social science as well. Visibility graph is a simple method to convert a fractal time series into a scale-free graph, and its structure has been shown to be related to the fractality (self-similarity) and complexity of the time series (Lacasa et al., 2008; Dick, 2012). The reliability of this methodology has been confirmed with extensive simulation of artificial fractal series and real (small) series concerning Gait disease Lacasa et al. (2009).

In a visibility graph each node of the graph represents a time sample of the time series, and an edge between two nodes shows the corresponding time samples can view each other. The visibility graph algorithm maps time series *X* to its Visibility Graph. Suppose the *ith* point of the time series is *X*_*i*_. Two vertices (nodes) of the graph, *X*_*m*_ and *X*_*n*_, are connected via a bidirectional edge if and only if the below equation is valid.

(1) $$\begin{array}{l} X_{m+j}<X_{n}+\left(\frac{n-\left(m+j\right)}{n-m}\right)\cdot \left(X_{m}-X_{n}\right) \end{array}$$

*where* ∀*j* ∈ *Z*^+^ *and j* < (*n* − *m*)

**Figure 1:**
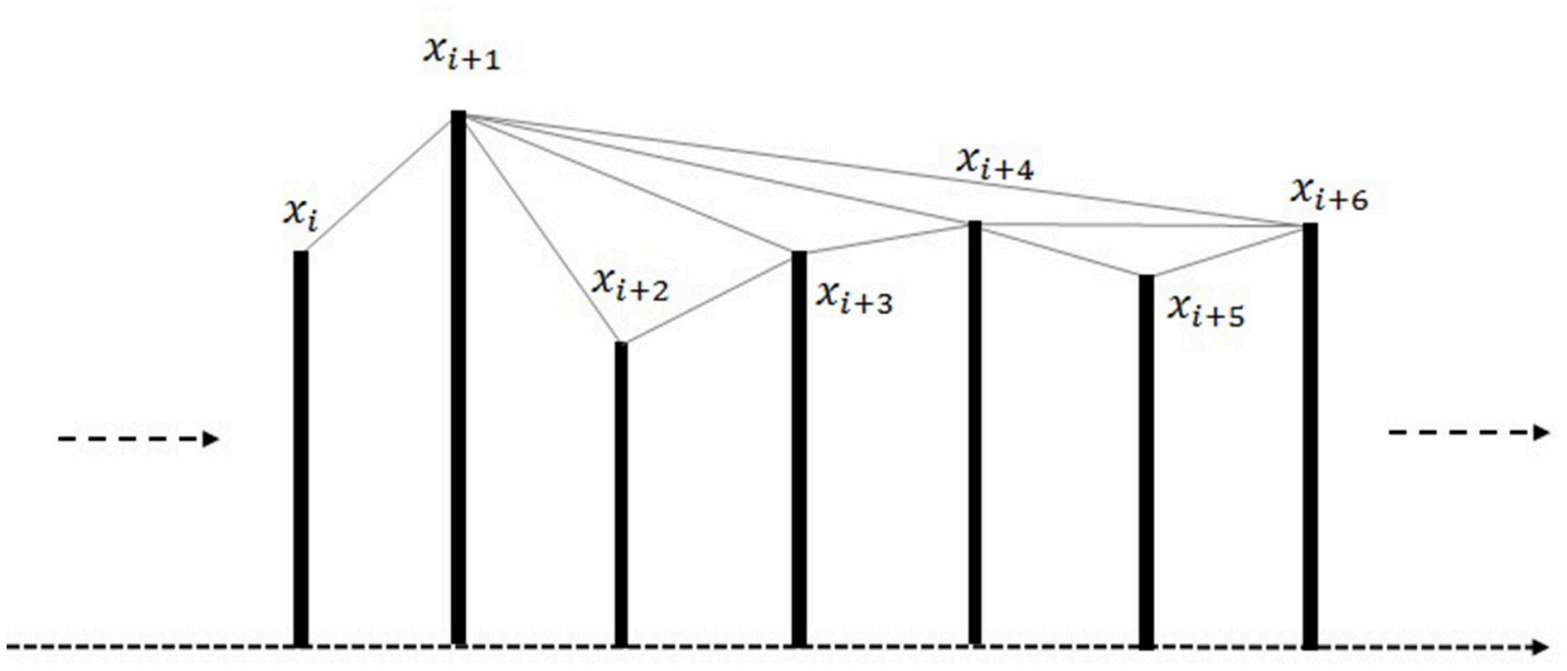
**Visibility Graph for time series X**.

As shown in Figure 1 *X*_*m*_ and *X*_*n*_ can see each other if the Equation 1 is satisfied. According to the logic the two sequential points of the time series can see each other hence all sequential nodes are connected together.

We should be converting the time series to positive planes as the above algorithm is valid for positive *X*-values in the time series.

As per the definition of a degree of a node in the graph - VG is the number of connections or edges the node has with other nodes. The degree distribution *P*(*k*) of overall network formed from the time series is then the fraction of nodes with degree *k* in the network. Thus if there are a total of *n* nodes the network and *n*_*k*_ of them have degree *k*, we have *P*(*k*) = *n*_*k*_∕*n*.

We say that two quantities satisfy the power law where one quantity varies as a power of another. The scale-freeness property of Visibility Graph states that the degree distribution of its nodes satisfies **Power Law** i.e., $P\left(k\right)=k^{-\lambda_{p}}$, where λ_*p*_ is a constant and it is known as **Power of Scale-freeness in Visibility Graph (PSVG)**. PSVG, denoted by λ_*p*_ here, which is calculated as the gradient of *log*_2_[*P*(*k*)] vs. *log*_2_[1∕*k*], corresponds to the amount of complexity as well as fractality of the time series, and also indicates the FD or the Fractal Dimension of the signal Lacasa et al. (2008, 2009). Ahmadlou et al. applied the visibility graph algorithm to convert time series to graphs, while preserving the dynamic characteristics such as complexity (Ahmadlou et al., 2010). The concept that each pattern and behavior in a fractal time series is repeated frequently, in different scales, has been proved by Ahmadlou et al. for visibility graph, by calculating the PSVG-s of a time series in multiple scale (Ahmadloua et al., 2012).

It has been proved that there exists a linear relationship between the PSVG- λ_*p*_ and the Hurst exponent H of the associated time series (Lacasa et al., 2009). Therefore, the visibility algorithm provides an alternative method to compute the Hurst exponent. Also it has been shown, for example, that gait cycle (the stride interval in human walking rhythm) is a physiological signal that displays fractal dynamics and long-range correlations in healthy young adults. The visibility algorithm predicts gait dynamics slow pace, in perfect agreement with previous results based on the usual method of detrended fluctuation analysis (Lacasa et al., 2009). The visibility graph is an algorithm that maps a time series into a graph. In doing so, classic methods of complex network analysis can be applied to characterize time series from a brand new viewpoint. Graph theory techniques can provide an alternative method to quantify long-range dependence and fractality in time series.

The present work is devoted to the comparative analysis of the Power of Scale-freeness of Visibility Graph (PSVG) of normal heart rate time-series during different meditation states.

## 3. Results

### 3.1. Data

We have taken the data from MIT-BIH Physionet database http://physionet.org/physiobank/database/meditation/data/ referred in the work done by Peng and Mietus (1999). The detailed description of the data is available in the website. It basically contains Heart Rate (Beats/minute) data recorded for pre-meditation and meditation states, for two groups of subjects performing Chi and Kundalini Yoga meditation respectively. The naming convention for the data followed in the subsequent texts of this paper are as per the website.

**Figure 2:**
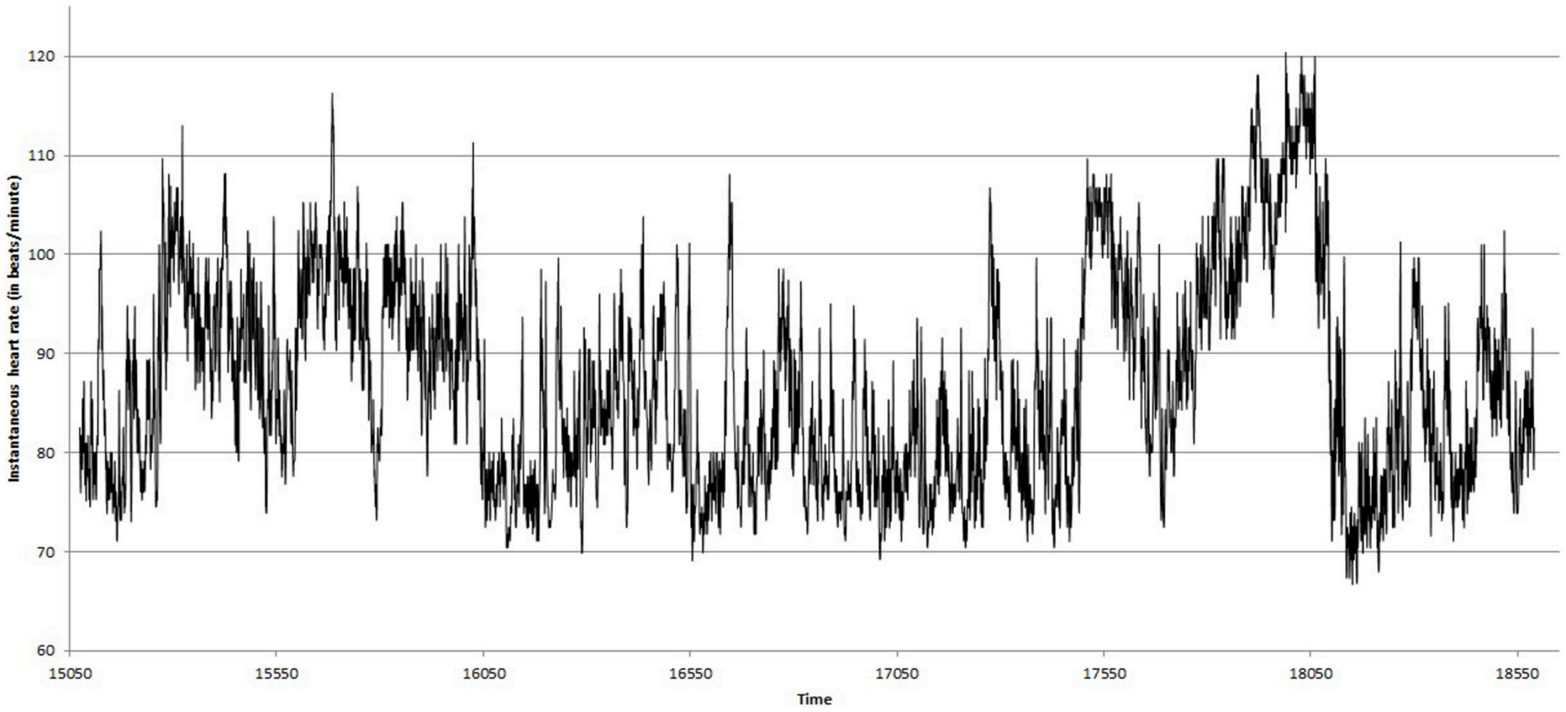
**Heart Rate (BPM) time series before the Chi meditation**.

**Figure 3:**
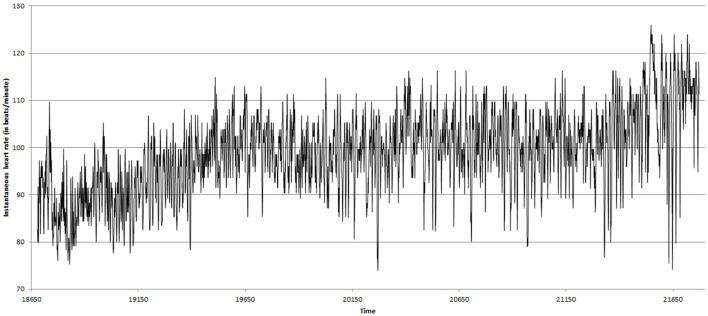
**Heart Rate (BPM) time series during Chi meditation, for the same subject**.

Figures 2, 3 show the heart beat time series of the subject C8(one of the eight subjects), before and during Chi meditation respectively.

It has been shown that the heart coherence can be a cardiac marker for the meditation states and also may be a general marker for the meditation state as the heart coherence is strongly correlated with heart rate variability (in terms of beats/min.-(BPM)) (Kim et al., 2013). Hence the present study of physical effects on meditation is focused on heart-rate dynamics. A recent article (Selvaraj et al., 2013) indicates that non-linear analysis on heart rhythm interval data can capture even finer emotional change. Moreover, in a few works, heart rate dynamics for meditation had been also been analyzed (Peng and Henry, 2004; Sarkar and Barat, 2008; Jiang et al., 2013).

### 3.2. Data analysis and results

Though Hurst exponent as mentioned in 2.3 has been used extensively and it has successfully detected long range correlations for different time-series, its computation is still a problem. The main problem about the Hurst exponent is the effect of **finite** data length for the estimation of Hurst exponent. The Hurst exponent yields to most precise and accurate result for random processes such as Brownian motion time-series with an **infinite** number of data points. But in real life situation we generally use finite time-series to estimate the Hurst exponent where long-range correlations in the time-series are partially broken into finite series and local dynamics corresponding to a particular temporal window are overestimated. Hence, the Hurst exponent calculated for shorter sample size inevitably deviates from its real value and higher the sample size the more precise is the result of the analysis. It is a common practice to use MF-DFA technique for this type of analysis due to its obvious advantage of having highest precision in the scaling analysis. However, as has been discussed earlier this method suffers from one lacuna. This theory demands that the length of the time-series to be analyzed has to be infinite, whereas in real life this time-series is always **finite** because there is no other option. In this regard another radically different rigorous method—**Visibility network analysis** (Lacasa et al., 2008, 2009) is extensively used over **finite** time-series data set and has produced reliable result in several domains of science and social science.

As already mentioned the advantage of visibility graph technique is that it gives more accurate estimate of Hurst exponent compared to other method (MFDFA) as the theory do not require an infinite series making this method a suitable for analysis **finite** time series (with less number of data points). The reliability of this novel and new methodology is confirmed with exhaustive numerical simulations as well as with analytic developments as mentioned in Section 2.4.

Therefore, we have applied both the **visibility graph** analysis as well as the **MF-DFA** analysis primarily because:

1. MF-DFA and Visibility Graph are two different methods for assessing the degree of complexity of a non-linear time series. Though their approaches are radically different, both of them are rigorous, robust and reliable among the methods proposed so far.
2. MF-DFA requires long time series which is not usually the cases in most real life phenomena. Visibility Graph on the other hand can give reliable and robust results even with very short time series.
3. We opted for the visibility graph technique and not mean Hurst exponent or DFA the mono-fractal parameter because as discussed it is a better technique for short sample sizes than any other parameters. There is also a mathematically proven relationship between the PSVG parameter with the Hurst exponent as mentioned in Section 2.4.
4. It is neither possible nor relevant to make a direct correspondence between the two methods. While one measures the degree of fractality the other measures the degree of multi-fractality. With both the methods having their own limitations our objective is to use the two methods and compare the results for the two states for the subjects separately and showcase a method of quantifying the physiological effects of meditation. Simultaneous analysis is beneficial just for double checking. If results obtained from one method follows the same pattern in a consistent fashion for the subjects with that of the other method we would like to justify the use of the two methods as a process for double-checking rather than cross-checking.

It is true that visibility network analysis has to be extended for tackling the multi-fractal behavior of time series which has not been possible so far though it needs mention that Ahmadloua et al. (2012) proposed analysis of PSVG values, based on averaging of fractality of time series in different scales. A generalization to handle multi-fractal behavior of a structure by PSVG technique is yet to develop.

#### 3.2.1. Analysis and results with MF-DFA

**Figure 4:**
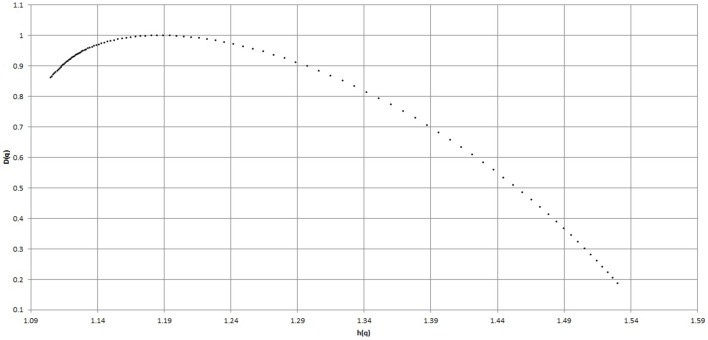
**Multi-fractal Spectrum during Chi meditation for the same subject**.

1. In our experiment (as described in Section 2.3) *s* varies from 16 as minimum to 256 as maximum value in log-scale.
2. The width of multifractal spectrum has been calculated for all the subjects, before and during Chi meditation of the subject C8.
3. Figure 4 shows the width of multifractal spectrum obtained from the BPM time series shown in Figure 2.
4. The widths of multifractal spectrum obtained for all the subjects are tabulated in Table 1 under MF-DFA columns.
5. Figure 9 represents the comparative study of the values of width of multifractal spectrum.

**Table 1:** **Pre and post meditation parameters as obtained by different techniques**.

	**PSVG**		**MF-DFA**		**Shuffled MF-DFA**	
**Subject**	**Meditation λ_*med*_**	**Pre-meditation λ_*pre*_**	**Meditation**	**Pre-meditation**	**Meditation**	**Pre-meditation**
C1	5.49	3.22	0.66	0.24	0.13	0.19
C2	3.83	2.95	0.43	0.21	0.08	0.09
C3	3.9	2.94	0.59	0.3	0.21	0.05
C4	3.96	2.61	0.45	0.39	0.05	0.02
C5	3.49	3.01	0.41	0.31	0.26	0.17
C6	3.89	3.12	0.28	0.28	0.14	0.14
C7	3.07	2.33	0.53	0.32	0.16	0.22
C8	3.89	3.45	0.68	0.43	0.09	0.11
Y1	5.19	1.42	−	−	−	−
Y2	3.25	1.9	−	−	−	−
Y3	3.6	2.28	−	−	−	−
Y4	1.94	1.32	−	−	−	−

#### 3.2.2. Analysis and results with visibility graph

**Figure 5:**
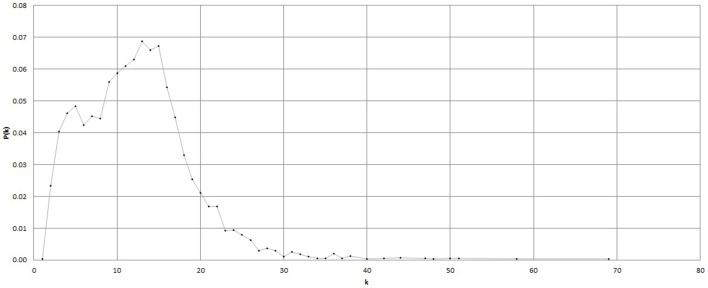
***P*(*k*) vs. *k* for the Heart Rate (BPM) time series during Chi meditation**.

**Figure 6:**
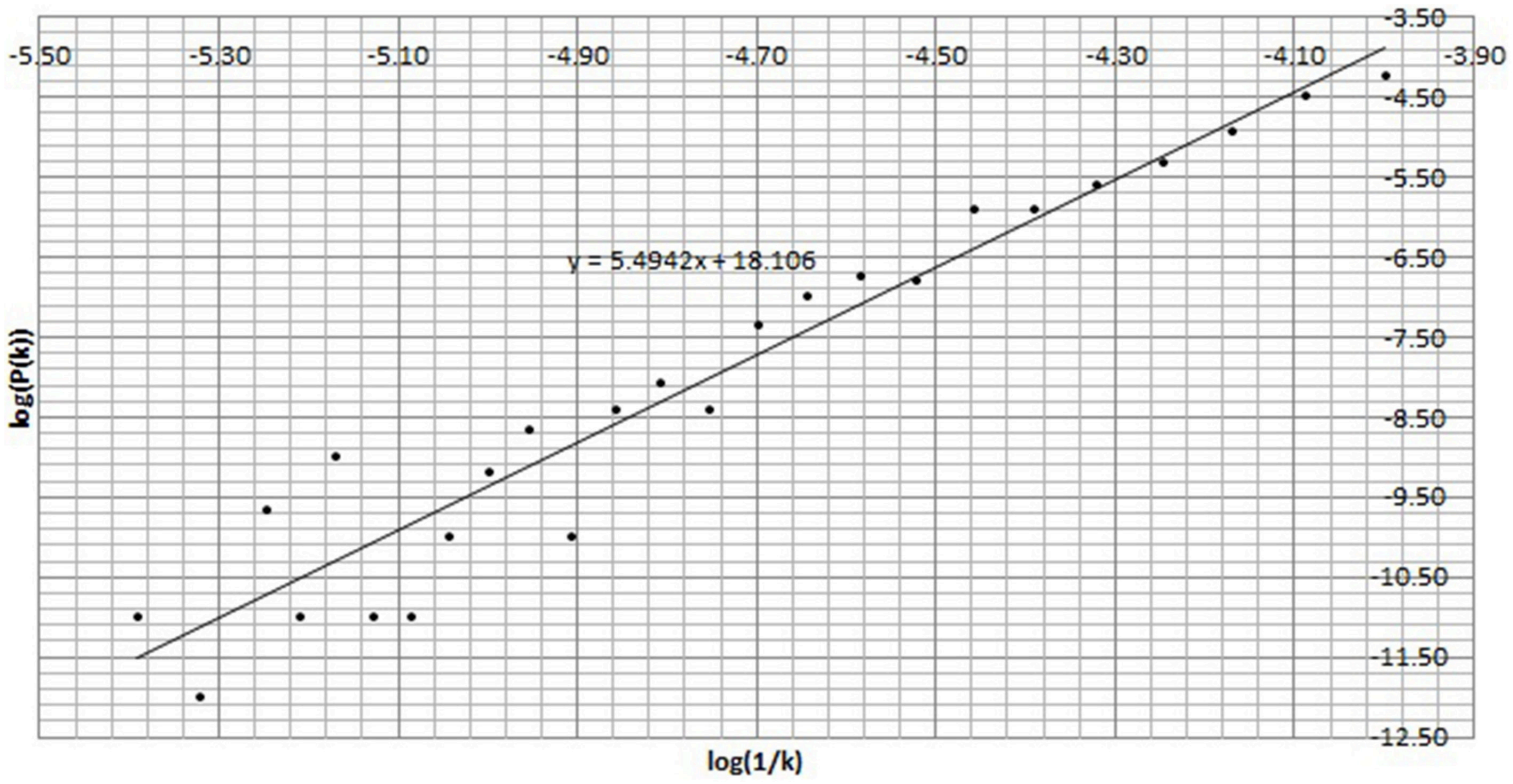
***log*_2_*P*(*k*) vs. *log*_2_(1∕*k*) for the Heart Rate (BPM) time series during Chi meditation**.

**Figure 7:**
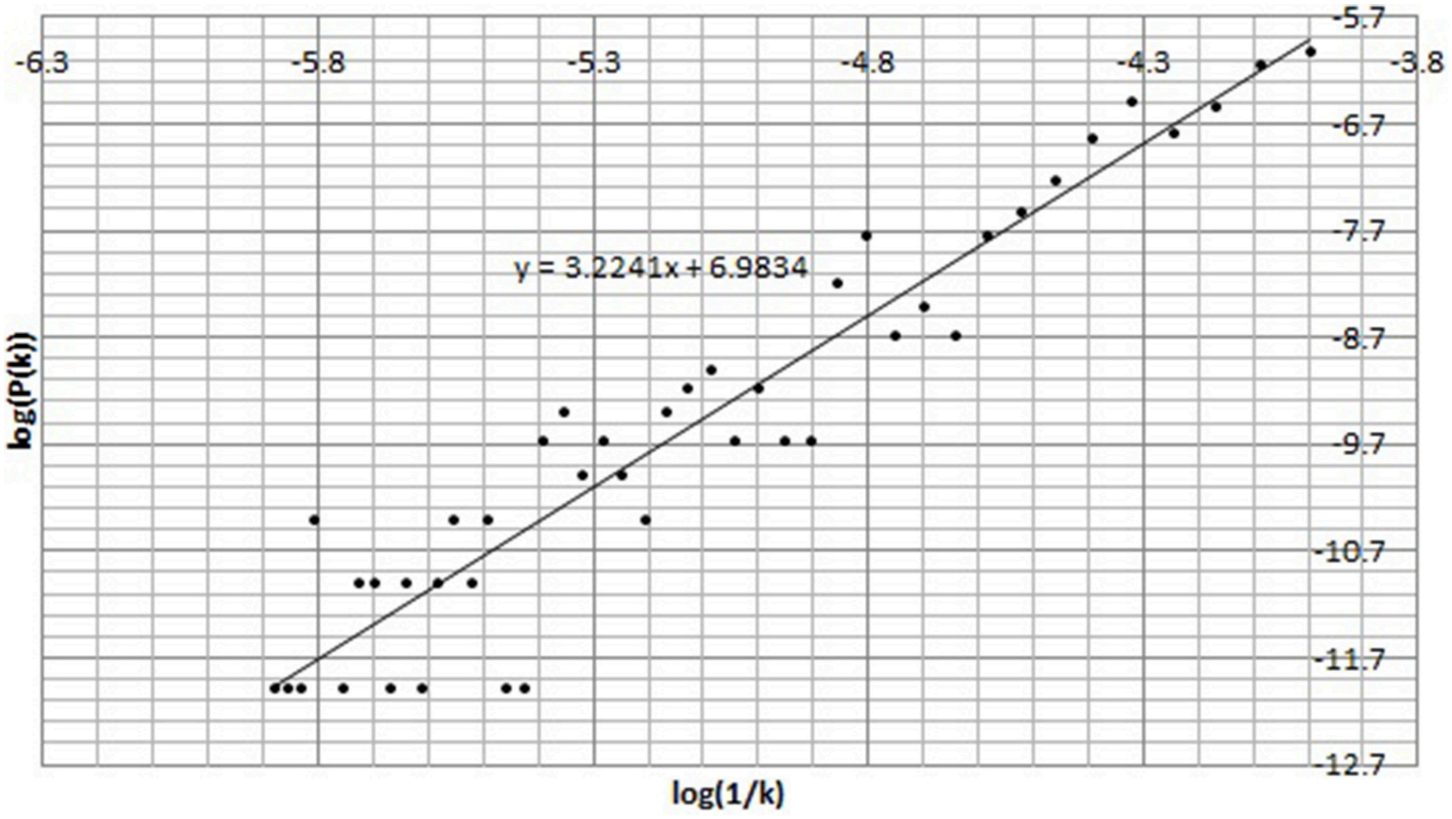
***log*_2_*P*(*k*) vs. *log*_2_(1∕*k*) for the Heart Rate (BPM) time series before Chi meditation**.

**Figure 8:**
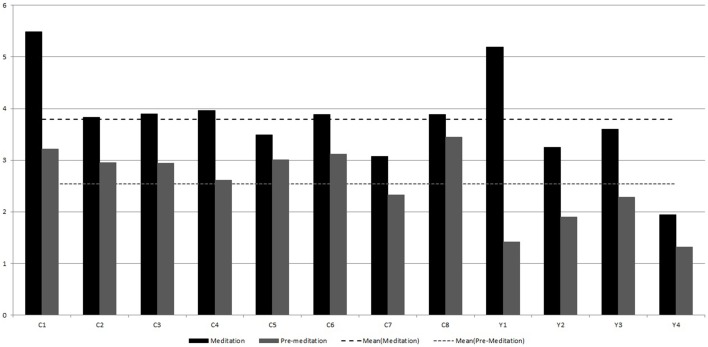
**Comparison of λ_*pre*_ and λ_*med*_ calculated from the Heart Rate (BPM) series for the two meditation groups**.

1. First *P*(*k*) w.r.t. *k* is calculated as per the method in Section 2.4. Figure 5 shows *P*(*k*) w.r.t. *k*, calculated from BPM time series of subject C8 during Chi-meditation. It is evident that it is confirming to the **Power Law**.
2. The the Power of Scale-freeness in Visibility Graph (PSVG) is represented by the gradient of *log*_2_[*P*(*k*)] vs. *log*_2_[1∕*k*] as shown in Figures 6, 7 for the same subject during and before meditation respectively.
3. These slopes are denoted by λ_*med*_ and λ_*pre*_ for meditation and pre-meditation states. They are calculated for all subjects and is tabulated in Table 1.
4. Figure 8 represents the comparative study of the PSVG values as given in Table 1.

## 4. Discussion

**Figure 9:**
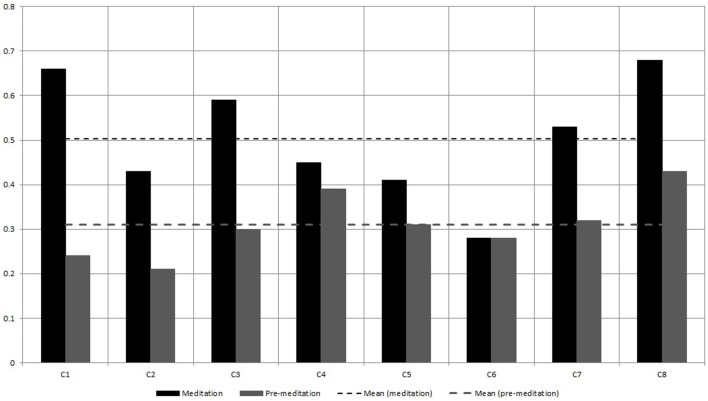
**Comparison of MF-DFA Spectrum width calculated from the Heart Rate (BPM) series for the two meditation groups**. We do not take cognizance for the Kundalini Yoga for MF-DFA as data size is too small.

Figures 8, 9 shows that the width of mutifractal spectrum and PSVG values (also tabulated in Table 1) are visibly different from each other for meditation and pre-meditation states.

### 4.1. General

1. We do not take serious cognizance of the results of MF-DFA analysis on the data for the Kundalini Yoga as the sample size is too low to give reliable results for MF-DFA analysis, where a large sample size is a prerequisite as discussed in Section 3.2. However, analysis on the data for Chi meditation is meaningful as the sample size is on the higher side for those subjects.
2. A simple correlation between the two methods for Chi-meditation is done to indicate a probable method of cross-checking. A positive correlation of 0.64 as a whole, 0.4 during meditation and 0.015 for pre-meditation is observed. However, analysis with bigger sample size with higher data points remains to be a scope for future work to produce a consistent cross-checking method.
3. The MF-DFA results are also obtained for the shuffled time-series obtained from the original time-series 1 to provide a baseline for MF-DFA values and in case of shuffled series the results clearly indicates that multi-fractal spectral widths are consistently lower in case of shuffled time-series compared to those of the original.
4. So far as visibility graph analysis is concerned there is no prerequisite for a large sample size. On the other hand, this method can give reliable results with as low as 400 data points (Jiang et al., 2013).
5. The PSVG values in most cases are more than 2 which indicates that the complexity of the time-series under investigation is on the higher side. Similar higher values of PSVG was reported by Lacasa et al. (2009).

### 4.2. Chi meditation

1. The widths of multifractal spectrum are consistently higher for meditation states for the Chi meditation group.
2. The widths of multifractal spectrum for individuals increases from 32.2 to 175% during meditation except for one case which shows no change.
3. The values of widths of multifractal spectrum are consistently < 0.5 in a pre-meditation scenario.
4. The Figure 8 shows that PSVG values during the meditation are always higher than the PSVG values for pre-meditation.
5. The PSVG values increase from 12.7 to 70.5% during the transition from pre-meditation to meditation states.

### 4.3. Kundalini yoga

1. PSVG values for the meditation state are consistently higher than the PSVG for pre-meditation state.
2. The overall mean values as shown with the dotted lines in Figure 8, have visible differences for meditation and pre meditation scenarios.
3. The PSVG values for individuals increases from 46.9 to 265.1% during the transition from pre-meditation to meditation states.

It is evident that the the degree of responses is different from pre-meditation to meditation states. Thus, we can conclude that two apparently distinct meditation protocol give rise to some physiological response. It might be interesting to Maity et al. (2015) where effect of music stimuli (drone) was studied with EEG signal using MF-DFA technique and it was shown that the effect of music had influenced the coefficient (the width multifractal spectrum) by 50%. It should be noted here that in this analysis the EEG data was used which is a manifestation of the function of the central nervous system. Whereas, the heart rate data is an outcome of the dynamics of the autonomic nervous system. Since the same method for the assessment of the degree of complexity was used and for us no meditation induced heart-beat (or ECG) data is readily available, we propose the perform this type of study in future works with larger datasets to arrive at a confident conclusion in absolute terms.

## 5. Conclusion

The results of this study are interesting and useful since the present investigation clearly indicates that meditation including Yoga significantly affects heart rate dynamics, and enhances the degree of complexity to be precise. The values obtained by PSVG analysis technique further hint at more pronounced effects during Kundalini Yoga.

Whereas, for the subject C6, MF-DFA technique did not show any increase of spectral width, but visibility technique yielded a 24.6% enhancement during meditation justifying the simultaneous use of two methods.

With the multi-fractal spectra being wider during meditation, we intend to conclude that a wider spectra may relate to a feeling of well being. However, the feeling of well-being is a very complicated phenomenon and there are more than one physiological parameters simultaneously playing their role toward feeling good. Chaos theory tells us that there is a natural background of chaos in our body—a loss of chaos can lead to an abnormal function—and in this respect meditation with a wider spectra leads to better functions of the body. To support this argument we may refer (Maity et al., 2015) where consistent increase of multi-fractal spectra were observed in EEG time series. This may confirm that increase of degree of complexity could be a parameter for quantification of well being and hence an improved therapy.

We may conclude that this analysis using two rigorous non-linear technique provide a method of double checking. Similar analysis should be done for other types of data for offering a benchmark in the assessment of cardiac states in a quantitative manner during different types of Yoga or meditation.

## Author contributions

All authors listed, have made substantial, direct and intellectual contribution to the work, and approved it for publication.

### Conflict of interest statement

The authors declare that the research was conducted in the absence of any commercial or financial relationships that could be construed as a potential conflict of interest.
